# mSphere of Influence: Structural Insights into the Molecular Mechanism Underlying Placental Malaria

**DOI:** 10.1128/mSphere.00391-21

**Published:** 2021-05-28

**Authors:** Maria del Pilar Quintana

**Affiliations:** a Centre for Medical Parasitology, Department of Immunology and Microbiology, University of Copenhagen, Copenhagen, Denmark

**Keywords:** *Plasmodium falciparum*, placental malaria, VAR2CSA structure, vaccine

## Abstract

Maria del Pilar Quintana works on immunology and pathogenesis of severe malaria. In this mSphere of Influence article, she reflects on how the papers “Structural basis for placental malaria mediated by Plasmodium falciparum VAR2CSA” (R. Ma, T. Lian, R. Huang, J. P. Renn, J. D. Petersen, J. Zimmerberg, P. E. Duffy, N. H. Tolia, Nat Microbiol 6:380–391, 2021, https://doi.org/10.1038/s41564-020-00858-9) and “Cryo-EM reveals the architecture of placental malaria VAR2CSA and provides molecular insight into chondroitin sulfate binding” (K. Wang, R. Dagil, T. Lavsten, S. K. Misra, C. B. Spliid, Y. Wang, T. Gustavsson, D. R. Sandoval, E. E. Vidal-Calvo, S. Choudary, M. O. Agerback, K. Lindorff-Larsen, M. A. Nielsen, T. G. Theander, J. S. Sharp, T. M. Clausen, P. Gourdon, A. Salanti, A. Salanti, Nat Commun 12:2956, 2021, https://doi.org/10.1038/s41467-021-23254-1) shed light on the precise structural details behind Plasmodium falciparum VAR2CSA binding to chondroitin sulfate A (CSA) in the placenta and how these novel insights have changed the way she will approach her work toward the discovery of new broadly cross-reactive/inhibitory antibodies targeting VAR2CSA.

## COMMENTARY

Malaria is a devastating parasitic disease caused by infection with protozoan parasites of the genus *Plasmodium*. There are an estimated 229 million malaria cases per year and 405,000 deaths, most of which are due to Plasmodium falciparum infection in Africa. Moreover, around 12 million pregnant women were exposed in 2019 to malaria infection in Africa alone, resulting in an estimated 822,000 children with low birth weight (1). Placental malaria (PM) is caused by the sequestration of the parasite-infected erythrocytes (IEs) in the vascular area of the placenta (the intervillous space); this binding has been associated with maternal anemia, decreased birth weight (due to fetal growth restriction), and preterm delivery (2). In PM, IE sequestration is mediated by the protein VAR2CSA, a member of the P. falciparum erythrocyte membrane protein 1 (PfEMP1) family that binds to chondroitin sulfate A (CSA), a glycosaminoglycan exclusively expressed in the placenta (3).

VAR2CSA is a large (∼350-kDa) transmembrane protein, transported by the parasite to the IE surface and containing a long and cysteine-rich extracellular portion composed of six Duffy binding-like (DBL) domains separated by four interdomain regions (IDs). Several domains have been implicated in VAR2CSA binding to CSA (4, 5), but the ID1-ID2a region, which includes DBL2X, seems to be sufficient for binding to CSA and is considered the minimal binding region (6, 7).

An important feature of PM in areas where malaria is endemic is that its prevalence decreases with increasing parity, suggesting that immunity against PM develops in an exposure-dependent manner, as is the case for clinical malaria in general. Protection has been correlated with increased levels of IgG antibodies targeting the surface of VAR2CSA-expressing IEs. The function of these antibodies seems to be blocking of the IE adhesion to CSA in the placenta, as higher levels of adhesion-blocking antibodies correlate with better pregnancy outcomes (e.g., increased maternal hemoglobin, higher birthweight and term delivery) (8–10). Several studies have reported that multigravidae living in areas where malaria is endemic acquire strain-transcendent antibody activity targeting parasites from different geographical locations and with different genetic background (8, 9), indicating that VAR2CSA can be exploited to protect against PM by vaccination. This is the rationale behind the two VAR2CSA-based vaccines currently in clinical trials: PAMVAC and PRIMVAC. Both vaccines are based on a recombinant, N-terminal VAR2CSA antigen containing the minimal binding region from a single parasite strain (FCR3 and 3D7, respectively). Disappointingly, both vaccines show very limited cross-reactivity and induce adhesion-blocking antibodies only against parasites expressing a VAR2CSA allele homologous to the one used for immunization (11, 12). Thus, the vaccines appear not to emulate the immunity acquired by natural exposure to VAR2CSA-expressing IEs. Several studies have demonstrated extensive interclonal sequence diversity among VAR2CSA variants, particularly those circulating in Africa (13–15). This could partially explain the inability of PAMVAC and PRIMVAC to induce a cross-inhibitory response. In addition, multiple studies indicate that regions other than ID1-ID2a contribute to VAR2CSA binding to CSA and that naturally acquired antibodies targeting these different regions also have adhesion-inhibiting properties (16–18).

High-resolution structures of DBL3X, DBL6ε, and DBL3X-DBL4ε have been determined, but only low-resolution structures of the full-length VAR2CSA have been available until the recent publication of the two papers discussed here (19, 20). This lack of knowledge has precluded a clear understanding of the host-parasite interactions mediated by VAR2CSA and its effective use as a target for vaccine development.

The two new papers present the first high-resolution (3- to 4-Å) cryo-electron microscopy (cryo-EM) structure of full-length VAR2CSA, both on its own and in complex with CSA (19, 20). The data indicate that VAR2CSA folds in a V/7-shaped structure, with the ID1-DBL2X-ID2-DBL3X-DBL4ε-ID3 segment forming a stable compact core (maintained by a high degree of interdomain interactions) followed by a flexible DBL5ε-DBL6ε arm. DBL1X is also structurally flexible and has only few interactions with the core structure (via DBL2X and DBL4ε). The core domains create a major and a minor CSA-binding channel, both containing positively charged residues that are highly conserved among different VAR2CSA alleles. The major binding site is discontinuous and is formed by a surface-exposed region of DBL2X and a hole located deep in the channel. This hole is formed by the NTS, DBL1X, DBL2X, and the DBL4ε domains. The smaller and separate minor binding channel is made up by residues in the C terminus of the DBL2X and the N terminus of ID2. Importantly, the structure of VAR2CSA in the presence or absence of CSA does not differ dramatically, and only minor differences are observed. The core seems to be stabilized, while the arm formed by DBL5ε-DB6ε becomes more flexible and possibly displaced upon binding to CSA. The two papers also address sequence variability, indicating that both the major and the minor CSA-binding sites are conserved among different VAR2CSA variants while the immediate flanking areas are not.

The data presented in these two papers constitute a breakthrough in our understanding of the molecular mechanism of VAR2CSA-mediated IE binding in the placenta, a parasite feature that ultimately leads to the development of PM. The data clearly show that several regions within the VAR2CSA sequence fold and coalesce to form the CSA-binding channel. The structure confirms the major participation of DBL2X and its flanking regions (the previously described minimal binding region) but also highlights the importance of other regions. In particular, the data underscore the NTS and DBL4ε domains as main components in the major CSA-binding site and of ID3 as the “glue” that maintains the molecule’s core structure, urging us to study these regions further and probably to include them in future vaccine design. The structural data accompanied by sequence conservation analysis also help us understand the failure of the current vaccine candidates to induce cross-reactive/inhibitory antibodies. Both candidates include only a portion of the binding site, probably having a negative impact on antibodies targeting this region, due to insufficient display of the functional epitopes involved in CSA binding. Additionally, the inclusion of variable regions (like those flanking the binding site) is likely to generate variant-specific antibodies rather than the desired cross-reactive antibodies.

The new structural data presented by the two papers (19, 20) have prompted me to reevaluate the strategy I am currently implementing for the discovery of new broadly cross-reactive and cross-inhibitory antibodies targeting VAR2CSA acquired during natural exposure to infection. Initially, my focus was on the use of the entire VAR2CSA minimal binding region (ID1-ID2) as the bait antigen to discover such antibodies. I am now looking deeper into the CSA-binding site, searching for conserved, functional, and surface-exposed regions beyond ID1-ID2 to be included in my bait antigens. I think that inclusion of such regions will improve the likelihood of successful identification and characterization of cross-reactive/inhibitory antibodies acquired during natural exposure to infection. These antibodies could have potential as prophylactic and/or adjunctive therapy in PM (argued in favor of in a recent review [21]) and will also support the rational design of future and improved VAR2CSA-based vaccines.
